# The Shell Crown

**Published:** 1906-04

**Authors:** R. M. Sanger

**Affiliations:** Orange, N. J.


### THE SHELL CROWN.

By R. M. SANGER, D. D. S., Orange, N. J.

(Read before the Second District Dental Society, State of New York, November, 1905.)

In making a shell crown three points are essential: 1st, a perfect fit at the neck of the tooth: 2d, a proper contour, and 3d, a perfect occlusion. In my hands the two piece crown can most expeditiously and accurately be made to meet these several requirements, and if you will bear with me for a little while I will give you the details of my procedure.

One of the most legitimate objections to the use of collars is the liability of impinging on the gum just under the gingivis, causing an irritation followed by the forming of a pus pocket and final exfoliation of the tooth root. This may be avoided by tucking a rope of cotton saturated with vapocaine under the gingivus to anesthetize the gum, then dissecting away the gum to about one thirty-second of an inch in depth; check the hemorrhage with adrenalin chloride 1/5000 and cauterize the wound with trichloracetic acid which gives a clear, dry eschar and gives you a sufficient depth for your collar without impinging on the gum.

The circumference of the neck is then taken with a dentimeter and a band of pure gold 30 gauge is cut the required length, annealed and bent around tooth with the joint at the buccal aspect, lapping the ends, which are then marked with an excavator. Remove the band, cut a trifle short of the mark and solder the ends with 20K. solder. Bevel the gingival edge and stretch the band back on the tooth with the joint at the palatal aspect, and with a small flat steel burnisher burnish the gold to fit the neck closely. Cut the band narrow enough to allow the patient to close the teeth without touching it and with a large ball burnisher, or pliers designed for that purpose, form the contour, while the collar is in position in the mouth. Then with the collar still in position, place a piece of softened modeling compound about the size of a hickory nut in the collar and allow the patient to bite; chill the compound and remove collar and compound together. Drop sticky wax in the under side, sticking the collar fast to the compound and chill. With a penknife or carving tool, carve out the cusps, press into mouldine, pour fusible metal die and swage 28 or 30 gauge gold, tack cusps to collar with the smallest particle of 18K. solder, and replace in mouth to correct bite, then solder and finish, and you should have a crown with perfect occlusion and so perfect an adaptation at the neck that the pressure exerted by the thumb and finger is sufficient to drive it to place. I do not think that there is ever any excuse for malleting either a collar or a crown to place.

Finally I have found it possible to get a closer adaptation of the cement by drilling a hole through the thickest part of the cusp and tapping it, fitting into this a piece of threaded gold wire. In setting the crown remove the gold wire and allow the surplus of cement to escape through the hole, and when the cement is nearly hard, clear it away from the hole and screw in the gold wire. As soon as the cement is thoroughly hard, cut the wire flush and polish.

I usually make and set these crowns at one sitting, but if two sittings are preferred, a little larger piece of modeling compound is used in taking the bite, giving a fair impression of the tooth on either side of the one to be crowned both above and below. This is thoroughly chilled, removed from the mouth and mounted on a crown articulator, a little oil or vaseline being placed on the gold band to admit of its ready removal from the cast. After separating a small piece of modeling compound is softened, pressed into the collar, and the plaster teeth closed into it, it is then chilled, and removed from the cast with the collar, and the balance of the work done as described above. This method has the advantage of two short sittings instead of one long one, the work being complete and ready to set on the arrival of the patient the second time. The ready made cusp forms that are furnished with the Hollingsworth and other crown systems can be employed in some cases instead of carving the cusps, the blank being selected and adjusted to the gold collar on the articulator. This is more expeditious than carving the cusps, but does not give so accurate an occlusion.—*Items.*

